# Influence of calcium sources on the bio-mineralization behavior of *Shewanella putrefaciens* and induced microbiologically influenced corrosion inhibition

**DOI:** 10.3389/fmicb.2025.1532151

**Published:** 2025-02-25

**Authors:** Yuntian Lou, Hao Zhang, Weiwei Chang, Jingzhi Yang, Xudong Chen, Xiangping Hao, Hongchang Qian, Dawei Zhang

**Affiliations:** ^1^Beijing Advanced Innovation Center for Materials Genome Engineering, Institute for Advanced Materials and Technology, University of Science and Technology Beijing, Beijing, China; ^2^National Materials Corrosion and Protection Data Center, University of Science and Technology Beijing, Beijing, China; ^3^Belt and Road Initiative (BRI) Southeast Asia Network for Corrosion and Protection (MOE), Shunde Innovation School, University of Science and Technology Beijing, Foshan, China; ^4^School of Materials Science and Hydrogen Energy, Foshan University, Foshan, China

**Keywords:** microbiologically influenced corrosion inhibition, microbial bio-mineralization, *Shewanella putrefaciens*, organic/inorganic calcium sources, carbon steel

## Abstract

The influence of different calcium sources on the mineralization behavior of *Shewanella putrefaciens* and their roles in microbiologically influenced corrosion inhibition (MICI) of Q235 carbon steel were investigated. Calcium lactate, calcium nitrate, and calcium L-aspartate were selected as alternative calcium sources to assess their effects on bacterial growth, carbonate deposition, and corrosion resistance. *S. putrefaciens* exhibited stable growth in all tested media, with the pH exceeding 8 after 14 days, promoting carbonate precipitation. Under sterile conditions, all calcium sources provided some corrosion inhibition, with calcium L-aspartate demonstrating the most effective protection. In bacterial inoculated systems, calcium lactate and calcium L-aspartate facilitated the formation of a continuous CaCO_3_ mineralized layer, significantly reducing corrosion, whereas calcium nitrate resulted in discontinuous carbonate deposits, promoting localized corrosion. Electrochemical impedance spectroscopy and potentiodynamic polarization analyses confirmed that the mineralized layers formed with calcium lactate and calcium L-aspartate significantly enhanced corrosion resistance, while calcium nitrate exacerbated corrosion due to nitrate-reducing bacterial activity. These findings emphasize the crucial role of calcium source selection in MICI and provide insights for optimizing microbial mineralization strategies for corrosion mitigation.

## 1 Introduction

Microorganisms are widespread across natural and industrial environments, where their activities significantly influence metal corrosion (Dong et al., 2022; Liu et al., 2023; Procópio, 2022; Xu et al., 2023; Zhou et al., 2022; Zuo et al., 2024). These microbes may either accelerate metal degradation through microbiologically influenced corrosion (MIC) or, alternatively, inhibit it in a process termed microbiologically influenced corrosion inhibition (MICI) (Zhang et al., 2023). As an environmentally sustainable approach to corrosion mitigation, MICI has attracted substantial research attention in recent years (Karn et al., 2020; Liu et al., 2022). Within MICI, microbial communities employ various mechanisms to counteract corrosion (Liu et al., 2024; Lou et al., 2021b), such as depleting corrosive agents, forming protective layers of extracellular polymeric substances (EPS), promoting microbial bio-mineralization, or secreting metabolic products that inhibit corrosion. Among these mechanisms, microbial bio-mineralization plays a critical role, as the mineralized layers formed act as stable, barrier-like coatings that provide long-lasting protection.

Microbial bio-mineralization serves as a key corrosion-inhibition mechanism within the MICI process. Using common carbon steel as an example, in oxic NaCl solution, electrochemical corrosion occurs, with oxygen reduction leading to the formation of iron oxides (e.g., Fe_2_O_3_) on the surface, while chloride ions promote localized corrosion, causing pitting and cracking. Microorganisms facilitate the deposition of mineralized coatings on the material surface, with carbonate deposition offering particularly strong protection (Fan W. et al., 2023; Guo et al., 2022). These mineralized layers effectively block the impact of environmental corrosive agents such as chloride ions and dissolved oxygen (Anbu et al., 2016; Little et al., 2007; Qin et al., 2020). Upon attachment to solid surfaces, microorganisms typically form biofilms, which consist of microbial cells embedded within an EPS matrix. This matrix, composed of organic macromolecules including cells, polysaccharides, proteins, and humic substances, acts as a natural adsorbent, attracting cations from the surrounding environment via electrostatic forces, complexation, and ion exchange (Flemming and Wingender, 2010; Sauer et al., 2022). Over time, these cations accumulate within the biofilm, and under appropriate conditions, mineralization is initiated (Guo et al., 2021; Shen et al., 2020). Because microbial mineralization is driven by the interaction of inorganic ions with organic components within the EPS, the resultant mineralized layers are dense and uniform, significantly enhancing the protection performance (Jonkers et al., 2010; Wang et al., 2014). Liu et al. (2018) demonstrated that marine *Pseudoalteromonas lipolytica* converts biofilms into biomineralized hybrid membranes of calcite and EPS in simulated marine environments, providing strong corrosion inhibition and self-healing properties. By comparing wild-type, EPS-overproducing, and EPS-deficient strains, they confirmed that increased EPS production enhanced corrosion resistance, linking effective biomineralization with EPS levels. Moreover, our previous study examined the corrosion inhibition of Q235 carbon steel by *Shewanella putrefaciens*-mediated calcium deposition in simulated seawater (Lou et al., 2024a). The results demonstrated that under calcium-rich conditions, *S. putrefaciens* rapidly formed a protective calcium carbonate layer, effectively blocking chloride diffusion and mitigating corrosion. Potentiodynamic polarization tests revealed that the presence of calcium decreased the corrosion current density, achieving an inhibition rate of 92.2% compared to the sterile condition. Electrochemical impedance spectroscopy (EIS) and scanning electrochemical microscopy (SECM) corroborated these findings. These results suggested that *S. putrefaciens* and its EPS adhered to the material surface, chelating Ca^2 +^ ions and establishing a localized environment (pH close to 8.5) conducive to carbonate precipitation, thereby synergistically suppressing corrosion through microbial respiration and calcium-induced mineralization.

Research indicates that the choice of calcium source plays a critical role in defining the quality of microbially induced mineralization products (Wang et al., 2024; Xue et al., 2022). For instance, in the well-established application of microbial bio-mineralization for concrete repair, microorganisms leverage their mineralization capability to fill cracks and bind fine particles, with the selection of calcium source markedly influencing the solidification and stability of the resulting mineralized layers (Zhang et al., 2024). While calcium sources with higher solubility ensure an adequate supply of Ca^2+^, their anionic components must be carefully selected to avoid inhibiting microbial metabolic activity or undermining mineralization. Organic calcium sources provide both Ca^2+^ and carbon, thus supporting microbial metabolism, whereas inorganic calcium sources generally produce more compact and stable mineralized products (Cui et al., 2023). Recent studies suggest that varying calcium sources significantly influence the crystal form, morphology, and size of calcium carbonate crystals (Fan Q. et al., 2023). In calcium chloride solutions, rhombohedral or egg-shaped calcite crystals ranging from 50 to 100 μm have been observed (Abo El Enein et al., 2012; Gorospe et al., 2013; Seifan et al., 2017), while calcium nitrate solutions yield smaller, spherical calcite crystals of 4–50 μm (Yi et al., 2021). Spherical vaterite and calcite crystals have also been identified in calcium acetate, calcium lactate, and calcium gluconate solutions, although crystal aggregation in organic calcium solutions complicates precise size measurement (Xu et al., 2015). To date, the effects of different calcium sources on the shape and size of calcium carbonate crystals remain unclear.

In this work, the introduction of chloride ions during mineral layer formation presents potential corrosion risks; therefore, we selected calcium lactate, calcium nitrate, and calcium L-aspartate as alternative calcium sources to study the effects of various organic and inorganic calcium sources on MICI induced by microbial bio-mineralization. Through detailed corrosion morphology analysis and electrochemical techniques, we systematically investigated how the choice of calcium source influences mineralization behavior induced by *S. putrefaciens*, providing valuable insights for optimizing microbial mineralization in diverse environmental applications.

## 2 Experimental methods

### 2.1 Materials, bacterium and culture medium

The Q235 carbon steel utilized in this study (composition: 0.1 C, 0.4 Mn, 0.12 Si, 0.02 S, 0.05 P, Fe balance, wt.%) was sectioned into coupons measuring 10 mm^3^ × 10 mm^3^ × 3 mm^3^. Each coupon was sequentially abraded using silicon carbide abrasive papers of 200, 400, 600, and 800 grit. The pretreatment procedure included ultrasonic cleaning in anhydrous ethanol, nitrogen drying, and UV sterilization, as described in a previous study (Lou et al., 2024b).

The *S. putrefaciens* strain (MCCC 1A02627) was sourced from the Marine Culture Collection of China (MCCC). For bacterial cultivation, 2216E medium was prepared with the following components: 5.0 g peptone, 1.0 g yeast extract, 0.1 g ferric citrate, 19.45 g NaCl, 5.98 g MgCl_2_, 3.24 g Na_2_SO_4_, 1.8 g CaCl_2_, 0.55 g KCl, 0.16 g Na_2_CO_3_, 0.08 g KBr, 0.034 g SrCl_2_, 0.08 g SrBr_2_, 0.022 g H_3_BO_3_, 0.004 g NaSiO_3_, 0.0024 g NaF, 0.0016 g NH_4_NO_3_, and 0.008 g NaH_2_PO_4_ all dissolved in 1 L of deionized water. The pH was adjusted to 7.2 ± 0.2. Three alternative media were also altered based on the standard 2216E medium by substituting CaCl2 with calcium lactate, calcium nitrate, and calcium L-aspartate, respectively, to ensure an equivalent calcium concentration. Both the media and equipment were sterilized at 121°C for 20 min in an autoclave (Panasonic, MLS-3781-PC) and subsequently subjected to 30 min of UV exposure. Using a hemocytometer and a light microscope (Zeiss, Lab A1) at 400× magnification, the initial concentration of planktonic *S. putrefaciens* was standardized to 10^6^ cfu/mL. A 100 mL Erlenmeyer flask was used for the sample immersion experiment, containing 50 mL of bacterial culture medium. The pretreated samples were placed at the bottom of the flask, which was then sealed with a breathable membrane at the opening. The flask was placed on a shaker operating at 50 rpm to ensure the coupon remained at the bottom of the flash. All bacterial cultivation experiments were conducted at 30°C. The growth of *S. putrefaciens* was monitored using a hemocytometer and a light microscope (Zeiss, Lab A1) at 400× magnification. Every 24 h, 100 μL samples were collected for continuous monitoring over a 14-day period.

### 2.2 Surface/interface analysis and weight loss measurements

The surface morphologies of the coupons following various immersion time were examined using scanning electron microscopy (SEM, FEI Quanta 250). Before conducting the SEM analysis, the surfaces of the coupons were dried in a nitrogen atmosphere to preserve their original morphologies. Furthermore, gold sputtering was carried out to improve surface conductivity. The product layer’s structure and composition (cross-section) were analyzed using SEM and energy dispersive spectrometry (EDS). Following nitrogen drying, the coupons were embedded in epoxy resin, abraded to expose the interface, and polished with 1,000 grit silicon carbide sandpaper. A gold layer was subsequently applied to enhance observation. Confocal laser scanning microscopy (CLSM, Keyence VK-X260 K) assessed the morphologies and surface roughness of the coupons after removing surface products, which were eliminated using fresh Clarke’s medium according to ASTM G1-03 guidelines (Wikie et al., 2014). The biofilm on the coupon surface was examined using CLSM (Leica, TCS SP8) following live/dead fluorescent staining. In this method, live cells fluoresced green and dead cells red, as a result of staining with SYTO-9 and propidium iodide (PI) dyes. The chemical composition of the surface product layers was analyzed using X-ray diffraction (XRD, D8 Advance, Bruker). Weight loss measurements were performed using an electronic balance (± 0.1 mg, Mettler Toledo). Coupons were weighed after cleaning and nitrogen drying, then immersed in the media under different conditions. Following immersion, surface products were removed as described, and the coupons were dried and reweighed. The weight loss for each media was calculated and recorded, with three parallel coupons analyzed per condition to ensure result reliability.

### 2.3 Electrochemical measurements

Electrochemical measurements were conducted using a Gamry Reference 600 Plus electrochemical workstation. The corrosion behavior of Q235 carbon steel was evaluated in different solutions employing a standard three-electrode configuration, where the Q235 specimen acted as the working electrode, a platinum foil was used as the counter electrode, and a saturated calomel electrode (SCE) served as the reference electrode. To ensure the accuracy and stability of the measurements, the open-circuit potential (OCP) was recorded for at least 10 min prior to each test. The linear polarization resistance (LPR) was measured at a scan rate of 0.125 mV/s within a potential window of −5 to 5 mV relative to *E*_*OCP*_. Potentiodynamic polarization curves were obtained after 14 days of immersion, with the potential range set from −250 mV to +250 mV vs. *E*_*OCP*_ and a scan rate of 0.166 mV/s. Electrochemical impedance spectroscopy (EIS) was conducted with a 5 mV AC signal over a frequency range from 10^5^ Hz to 10^–2^ Hz. EIS data were analyzed using ZSimDemo software. All experiments were repeated on three parallel coupons to ensure reproducibility.

## 3 Results and discussion

**FIGURE 1:**
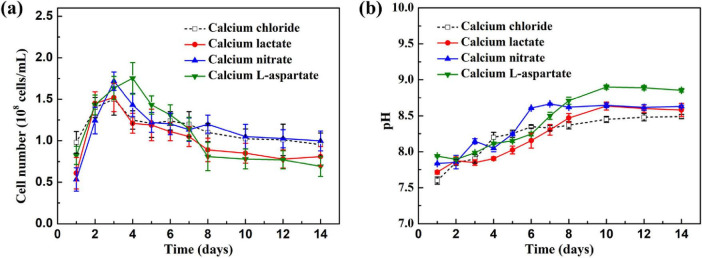
**(A)** Growth curves of *S. putrefaciens* in culture with different calcium source over time; **(B)** the change of pH values in culture with different calcium source over time.

Figure 1 illustrates the growth characteristics of *S. putrefaciens* under different calcium sources. In Figure 1A, the growth curves of *S. putrefaciens* under various conditions are shown. Throughout the immersion period, *S. putrefaciens* in media with different calcium sources exhibited two distinct growth phases. During the initial 3 days, rapid proliferation was observed. Subsequently, the growth rates gradually slowed. Throughout the immersion cycle, media with calcium lactate, calcium nitrate, and calcium-L-aspartate as calcium sources effectively maintained a relatively stable growth state for *S. putrefaciens*, indicating that the replacement of the above three calcium sources had no effect on the growth of *S. putrefaciens*. Figure 1B shows the pH variation over time for media with different calcium sources. During the first seven days of immersion, the pH values of 2216E media with various calcium sources tended to increase, stabilizing above pH 8.5 in the latter half of the immersion period. The pH level above 8.5 in the later stages of culture met the requirements for carbonate deposition (Carré et al., 2020).

**FIGURE 2:**
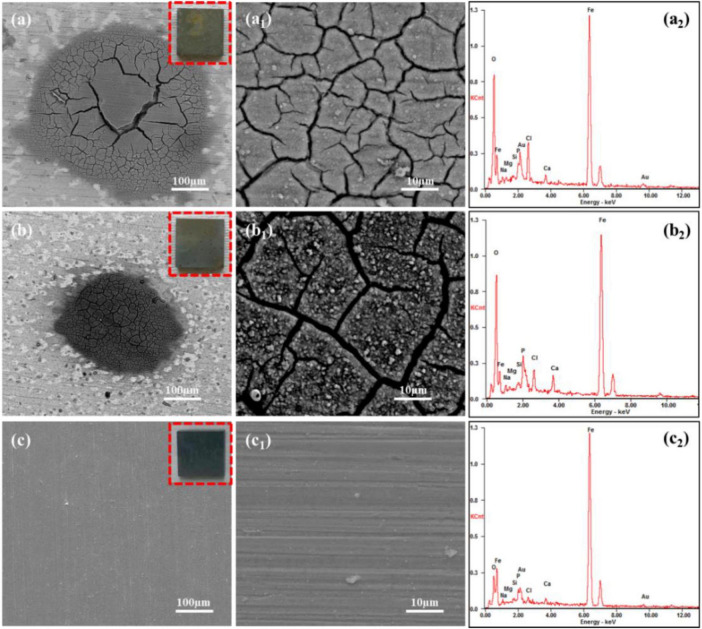
SEM and EDS analysis of corrosion products on Q235 carbon steel coupons after 14 days of immersion in sterile media with different calcium sources: **(A–A_2_)** calcium lactate, **(B–B_2_)** calcium nitrate, **(C–C_2_)** calcium L-aspartate.

Figure 2 shows the surface morphology and EDS analysis of Q235 carbon steel coupons after 14 days of immersion in sterile 2216E medium with calcium lactate, calcium nitrate, and calcium-L-aspartate as calcium sources. In Figure 2A, under the calcium lactate condition, the macroscopic image within the red box revealed that the coupon surface was mainly covered by gray corrosion products, with randomly distributed clusters of brownish-yellow localized corrosion products. SEM analysis indicated that these clusters resulted from localized corrosion, leading to minor accumulations of corrosion products. Upon higher magnification, the surface of these clusters showed a plaque-like, cracked morphology. The EDS results indicated significant amounts of Fe and O in these clusters, suggesting that iron oxides were the main components. Additionally, elements such as Cl, P, Mg and Na were detected, indicating that the corrosion product clusters were relatively loose and adsorbed inorganic salts from the medium. However, areas outside these corrosion clusters showed no severe corrosion, and faint polishing marks from sandpaper remained visible. Figure 2B shows the surface morphology and composition of corrosion products formed on Q235 carbon steel after 14 days of immersion in 2216E medium with calcium nitrate as the calcium source. The surface exhibited irregularly distributed clusters of dark brown corrosion products. Around these localized corrosion clusters, the surface showed no significant corrosion, and the overall corrosion layer was relatively thin, with some areas showing partial detachment. Upon further magnification of the dark brown corrosion clusters (Figure 2B1), the surface appeared rough, loose, and cracked. EDS analysis revealed that the primary components of these corrosion clusters were Fe and O, similar to the calcium lactate medium, with additional inorganic salts adsorbed from the medium. Figure 2C shows the surface morphology and composition analysis of Q235 carbon steel after 14 days of immersion in sterile 2216E medium with calcium-L-aspartate as the calcium source. The macroscopic image (red box in Figure 2C) showed no apparent signs of corrosion. Upon higher magnification, the surface appeared smooth and flat, with visible polishing marks from sandpaper. The corresponding EDS analysis revealed a strong Fe signal with relatively weak signals for other elements, and no prominent O peak was detected. Given that the detection depth of EDS is typically between 100 nm and 1 μm, it was inferred that a nanoscale adsorption film had formed on the coupon surface, effectively inhibiting corrosion (Zhang et al., 2020).

**FIGURE 3:**
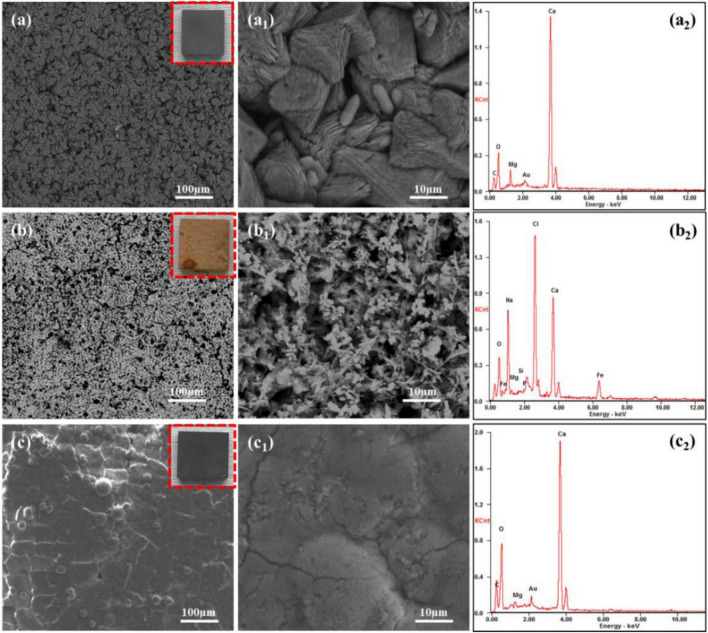
SEM and EDS analysis of corrosion products on Q235 carbon steel coupons after 14 days of immersion in *S. putrefaciens*-inoculated media with different calcium sources: **(A–A_2_)** calcium lactate, **(B–B_2_)** calcium nitrate, **(C–C_2_)** calcium L-aspartate.

Figure 3 presents the surface morphology and EDS composition analysis of Q235 carbon steel coupons after 14 days of immersion in 2216E medium with different calcium sources in inoculated media. In Figure 3A, the macroscopic image showed that the surface of the carbon steel coupon using calcium lactate as the calcium source was uniformly covered with a smooth, dark gray mineralized layer, with no visible corrosion. SEM observations reveal the surface was densely packed with rhombohedral mineralized particles. Compared to the mineralized layer formed in standard 2216E medium (Lou et al., 2021a; Lou et al., 2023), these particles retained their triangular and rhombohedral shapes, but with more rounded edges. Further magnification reveals cylindrical micro-particles between the rhombohedral particles, which may represent early-stage in-situ mineralization of *S. putrefaciens* cells. The corresponding EDS analysis (Figure 3A2) showed that the main elements on the surface are C, O, Ca, and Mg, indicating that the mineralized particles were primarily composed of CaCO_3_. In Figure 3B, the Q235 carbon steel coupon immersed for 14 days in inoculated medium with calcium nitrate exhibited significant corrosion, with extensive yellow-brown corrosion products on the surface. Localized blistering and rust layer detachment indicated that these products were relatively loose. SEM analysis revealed numerous pores on the loose rust surface, with rod-shaped bacteria embedded within the corrosion products (Figure 3B1). EDS analysis detected a high concentration of Cl, likely from the NaCl in the culture medium, suggesting that the loose corrosion layer promotes the accumulation of inorganic salts, increasing susceptibility to localized corrosion. Additionally, substantial amounts of Ca were detected, although it remains unclear from the morphology whether calcium deposition has occurred, and further tests will be conducted. Figure 3C presents the surface morphology and composition of a Q235 carbon steel coupon after 14 days of immersion in the inoculated medium with calcium-L-aspartate. The macroscopic image (red box in Figure 3C) showed a dark gray product film uniformly covering the surface, with a noticeable loss of metallic luster compared to the sterile group. SEM observations revealed a slate-like morphology with fine surface cracks. Composition analysis indicated that the main elements in the surface products were C, O, and Ca, suggesting that, under the calcium-L-aspartate condition, *S. putrefaciens* could still induce a relatively dense CaCO_3_ deposition layer on the carbon steel surface. These findings indicated that using calcium nitrate as the calcium source altered the corrosion inhibition behavior of *S. putrefaciens* on Q235 carbon steel, promoting corrosion instead. In contrast, in the medium with calcium lactate or calcium-L-aspartate, *S. putrefaciens* could still form a complete CaCO_3_ mineralized layer, although the morphologies of these layers differed from those in the standard 2216E (Lou et al., 2024a).

**FIGURE 4:**
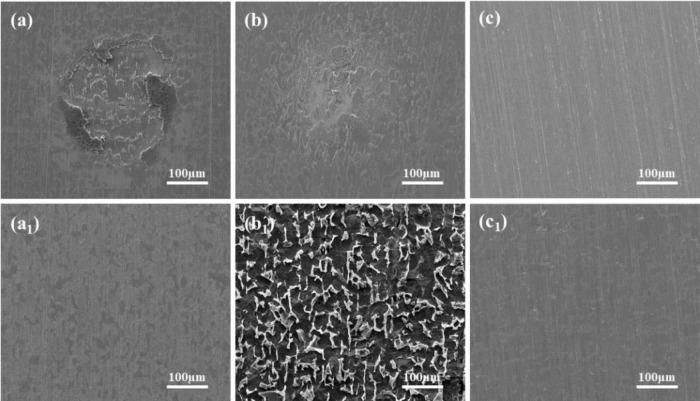
Surface morphology of carbon steel coupons after 14-day immersion and corrosion product removal in sterile and *S. putrefaciens*-inoculated media: **(A,A_1_)** calcium lactate, **(B,B_1_)** calcium nitrate, **(C,C_1_)** calcium L-aspartate.

As shown in Figure 4, the morphology of Q235 carbon steel coupons, after 14 days of immersion in media with different calcium sources under both inoculated and sterile conditions, is presented, with corrosion products removed. Under sterile conditions (Figures 4A, B), the coupons immersed in calcium lactate and calcium nitrate media exhibited visible localized corrosion signs. In contrast, the coupon in calcium-L-aspartate medium showed no significant corrosion, with only minor localized corrosion traces observed along the scratch marks. In the inoculated media, the mineralized layers induced by calcium lactate and calcium-L-aspartate effectively mitigated corrosion on the carbon steel coupons, showing only slight corrosion traces on the coupon surface after 14 days of immersion. Notably, severe corrosion was observed on the carbon steel surface in the calcium nitrate medium, displaying a characteristic pattern where localized corrosion progressively transitioned to uniform corrosion.

**FIGURE 5:**
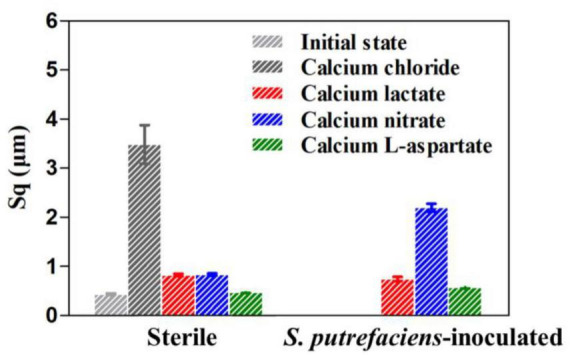
Surface roughness of Q235 carbon steel coupons after removal of surface products under different conditions for 14 days.

Due to the similarity in corrosion morphology across different treatments, which makes precise evaluation and analysis challenging, surface roughness (Sq, root mean square height) was measured to further compare the corrosion levels of the coupons, as shown in Figure 5. In the calcium chloride medium, the surface roughness of the coupons increased nearly sevenfold after 14 days of immersion compared to their initial state (Lou et al., 2024a). However, regardless of the presence of *S. putrefaciens*, the coupons in calcium-L-aspartate medium exhibited the lowest Sq values, followed by those in calcium lactate. Under sterile conditions, the Sq values of coupons in calcium nitrate and calcium lactate media were relatively close. Combined with the morphological observations, this suggests that both calcium nitrate and calcium lactate media exert some inhibitory effect on corrosion. In the inoculated medium, however, the Sq value of the coupon in calcium nitrate medium was significantly higher than in the other groups. This may be due to the nitrate-reducing respiratory activity of *S. putrefaciens*, which accelerates electron transfer between the bacteria and the carbon steel surface, thereby accelerating corrosion (Xu et al., 2013).

**FIGURE 6:**
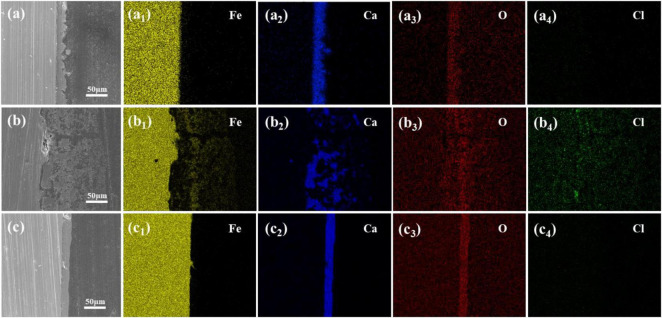
Cross-sectional morphologies and elemental distributions of microbial-induced product layers after 14 days under different calcium sources: **(A–A4)** calcium lactate, **(B–B4)** calcium nitrate, and **(C–C4)** calcium L-aspartate.

The results above indicate that changing the calcium source in sterile media has minimal impact on Q235 carbon steel coupons and may even inhibit corrosion. However, in the presence of *S. putrefaciens*, different calcium sources lead to product layers with distinct morphological characteristics on the carbon steel surface. To further analyze the effect of these product layers on the corrosion behavior of carbon steel, cross-sectional morphology and EDS elemental distribution analyses were conducted on coupons immersed for 14 days in media with calcium lactate, calcium nitrate, and calcium-L-aspartate as calcium sources, as shown in Figure 6. In all calcium-source conditions, a significant amount of Ca was detected in the product layer on the coupon surface, though the morphologies differed markedly. The mineralized layer in the calcium lactate medium was relatively dense with uneven surface undulation, completely covering the surface of carbon steel, and Cl is not detected at the interface (Figures 6A–A4). The mineralized layer formed in the calcium-L-aspartate medium also showed a dense structure (Figures 6C–C4). Compared to the calcium lactate condition, the layer in the calcium-L-aspartate medium exhibited less surface undulation, and similarly, no Cl enrichment was observed. In contrast, cross-sectional analysis of the coupon in the calcium nitrate medium revealed that granular mineralized particles were dispersed within the rust layer in a discontinuous pattern, with loose adhesion to the carbon steel substrate. This structure was likely to have caused uneven distribution of dissolved oxygen, leading to the formation of oxygen concentration cells. EDS analysis showed a high concentration of Cl within the rust layer, with localized accumulation at the interface. Figure 6B1 highlighted noticeable localized corrosion on the coupon surface, further supporting this observation.

**FIGURE 7:**
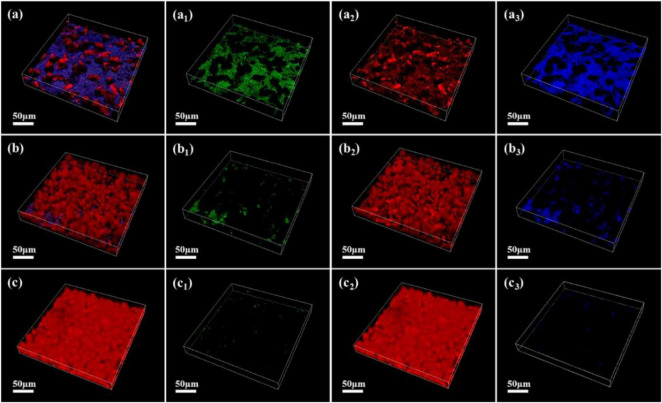
CLSM images of biofilms on the surface of Q235 carbon steel coupons in 2216E medium containing calcium lactate at different immersion times: **(A–A_3_)** 3 days; **(B–B_3_)** 7 days; **(C–C_3_)** 14 days.

**FIGURE 8:**
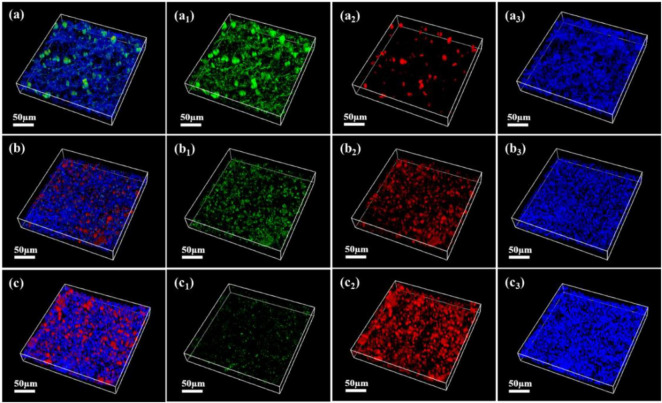
CLSM images of biofilms on the surface of Q235 carbon steel coupons in 2216E medium containing calcium nitrate at different immersion times: **(A–A_3_)** 3 days; **(B–B_3_)** 7 days; **(C–C_3_)** 14 days.

**FIGURE 9:**
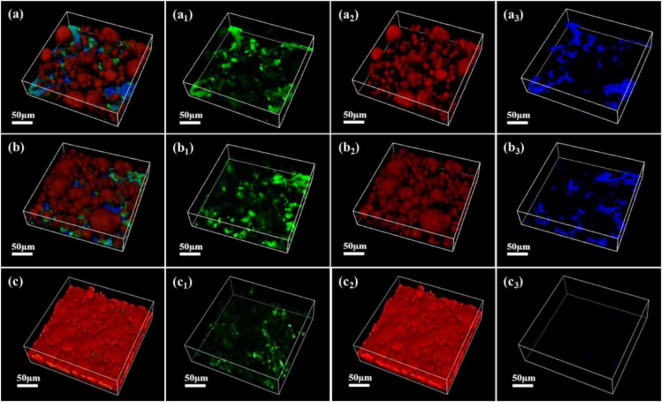
CLSM images of biofilms on the surface of Q235 carbon steel coupons in 2216E medium containing calcium-L-aspartate at different immersion times: **(A–A_3_)** 3 days; **(B–B_3_)** 7 days; **(C–C_3_)** 14 days.

As shown in Figures 7–9, the changes in biofilm formation on coupon surfaces with calcium lactate, calcium nitrate, and calcium-L-aspartate as calcium sources are illustrated. Green fluorescence represents live bacteria, red represents dead bacteria, and blue indicates reflection from the substrate. By the third day of immersion, numerous live bacteria had attached to the carbon steel surface and formed biofilms across all calcium-source conditions. As immersion time progressed, the number of live bacteria gradually decreased, replaced by the growth of mineralized particles. In the initial stage of mineralization induced by *S. putrefaciens*, bacterial cells acted as nucleation sites, subsequently becoming encased by smaller mineralized particles, which ultimately led to cell death as nutrient exchange with the external environment was impeded. As the mineralized particles grew, they became much larger than the bacterial cells. Suspended bacteria in the medium continued to adhere to the surfaces of the mineralized particles, repeating the mineralization process. Consequently, in Figures 7–9, fluorescence images of dead bacteria showed the gradual enlargement of mineralized particles with embedded dead cells, making their morphology increasingly visible. The blue reflection from the carbon steel matrix clearly indicated the extent of mineralized layer coverage on the coupon. By day 14, the blue reflection on the surface of coupons in calcium lactate and calcium-L-aspartate media had disappeared completely, indicating full coverage by the mineralized layer. In contrast, the surface of coupons in the calcium nitrate medium displayed a loose morphology with corrosion products. These findings, consistent with the SEM results of the coupon interfaces, demonstrated that the mineralized layers formed in calcium lactate and calcium-L-aspartate media effectively inhibited corrosion of the substrate. Although calcium nitrate as a calcium source did not prevent mineralization, the resulting mineralized particles lacked corrosion-inhibiting properties for the substrate.

**FIGURE 10:**
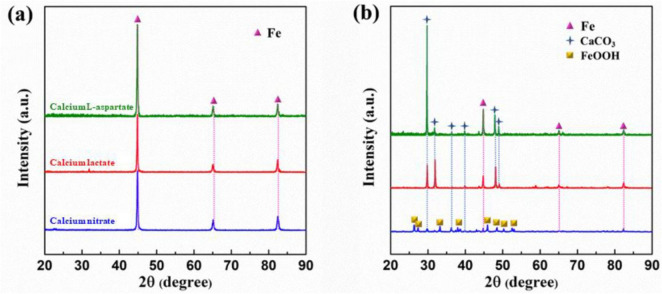
XRD analysis of surface products on Q235 carbon steel coupons after 14 days of immersion in sterile **(A)** and inoculated **(B)** media with different calcium sources.

As shown in Figure 10, XRD results are presented for Q235 carbon steel coupons immersed for 14 days in calcium lactate, calcium nitrate, and calcium-L-aspartate media under both sterile and inoculated conditions. In the sterile media, signal peaks were detected at 44.7°, 65.1°, and 82.4° for all three calcium sources. Based on SEM morphology and EDS composition analysis, these peaks corresponded to elemental iron, as the corrosion product layer on the coupon surface in the sterile group was relatively thin, allowing only the characteristic peaks of iron to be detected by XRD. In contrast, in the bacterial group, XRD diffraction peaks in calcium lactate and calcium-L-aspartate media indicated that the mineralized layer on the carbon steel surface consisted of CaCO_3_, with characteristic peaks appearing at 29.5°, 37°, and 47.9°. For the calcium nitrate inoculated condition, the overall signal intensity was weaker, with detected peaks for FeOOH, CaCO_3_, and faint traces of elemental Fe, suggesting a lower crystallinity of the corrosion products on the coupon surface (de la Fuente et al., 2006). This result indicated that, in the presence of *S. putrefaciens* and high concentrations of calcium nitrate, corrosion of the carbon steel coupons was exacerbated.

**FIGURE 11:**
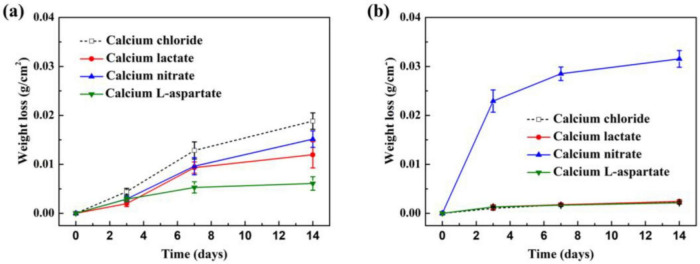
Corrosion weight loss of Q235 carbon steel coupons after different immersion times in sterile **(A)** and inoculated **(B)** media with various calcium sources.

As shown in Figure 11, weight loss measurements of Q235 carbon steel coupons in sterile and bacterial media with different calcium sources were conducted. Figure 11A displays the weight loss trends under sterile conditions, where the corrosion-induced weight loss of carbon steel coupons increases with immersion time. Compared to the standard 2216E medium, the calcium-L-aspartate medium exhibited the most significant corrosion inhibition effect on the carbon steel coupons, with a weight loss of only 0.0061 g/cm^2^ after 14 days, representing just 32.4% of the control group. The weight loss trends in calcium lactate and calcium nitrate media were similar, with both reaching approximately 0.01 g/cm^2^ by day 7. After extending the immersion period to 14 days, the weight loss in the calcium nitrate group reached 0.0151 g/cm^2^, while that in the calcium lactate condition was 0.0124 g/cm^2^. These results indicated that in the absence of bacteria, calcium lactate, calcium nitrate, and calcium-L-aspartate all exhibited some corrosion inhibition effect on carbon steel coupons, with calcium-L-aspartate demonstrating the most pronounced effect, consistent with SEM results presented earlier. Figure 11B shows the weight loss trends in inoculated media. It was evident that, except for the calcium nitrate condition, the presence of *S. putrefaciens* significantly inhibited the corrosion process of carbon steel. In both calcium lactate and calcium-L-aspartate media, the weight loss of the coupons remained almost unchanged after day 3 of immersion, indicating stable corrosion inhibition. In contrast, in the calcium nitrate medium, the weight loss of Q235 carbon steel increased substantially, reaching 0.0229 g/cm^2^ by day 3, which exceeded the weight loss observed in any sterile condition by day 14. Although the rate of weight loss slowed after 7 days, likely due to the formation of a corrosion product layer comprising FeOOH, CaCO_3_, and EPS as indicated by the XRD results, the corrosion rate remained higher than in the other media. By the end of the 14-day immersion period, the weight loss in the calcium nitrate medium reached 0.0315 g/cm^2^.

**FIGURE 12:**
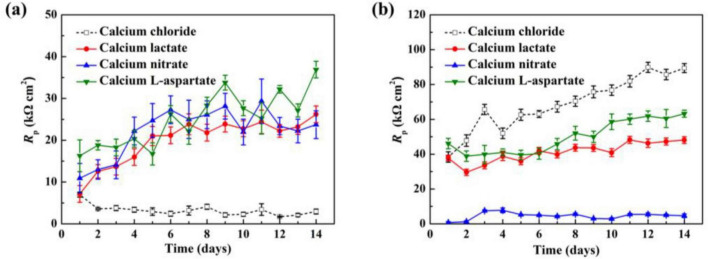
The *R*_p_ values of Q235 carbon steel coupons after different immersion times in sterile **(A)** and inoculated **(B)** media with various calcium sources.

As shown in Figure 12, linear polarization resistance (*R*_*p*_) measurements were conducted on Q235 carbon steel coupons in sterile and inoculated media with different calcium sources, tracking *R*_*p*_ values over time. In the sterile media (Figure 12A), *R*_*p*_ values in the standard 2216E medium remained consistently low. However, with different calcium sources, the *R*_*p*_ values of the carbon steel coupons increased overall, consistent with the weight loss data presented earlier. The trends in calcium lactate and calcium nitrate media were similar, showing two distinct phases: a rapid increase in *R*_*p*_ during the first 4 days of immersion, with values rising from 7.1 to 15.9 kΩ⋅cm^2^ in the calcium lactate medium and from 10.9 to 22.2 kΩ⋅cm^2^ in the calcium nitrate medium, more than doubling in each case. Following this phase, *R*_*p*_ values showed a steady and gradual increase until the end of the immersion period, reaching 26.2 kΩ⋅cm^2^ and 23.7 kΩ⋅cm^2^, respectively, by day 14. The *R*_*p*_ values in bacterial media are shown in Figure 12B. In the control 2216E medium, the *R*_*p*_ values of carbon steel coupons remained the highest across all groups. In contrast, the *R*_*p*_ values in calcium lactate and calcium-L-aspartate media were lower than those in the control group but still showed a continuous upward trend over time. Under the calcium nitrate condition, bacterial corrosion behavior accelerated, as indicated by consistently low *R*_*p*_ values throughout the immersion period.

Based on these results, calcium lactate, calcium nitrate, and calcium-L-aspartate exhibited corrosion-inhibiting effects on Q235 carbon steel coupons in the absence of bacterial involvement. However, when *S. putrefaciens* was present, only the carbonate mineralized layers formed under calcium lactate and calcium-L-aspartate conditions effectively inhibited corrosion on the carbon steel coupons. In contrast, in the calcium nitrate medium, the addition of nitrate ions enhanced the nitrate-reducing respiratory activity of *S. putrefaciens*, ultimately leading to accelerated corrosion.

**FIGURE 13:**
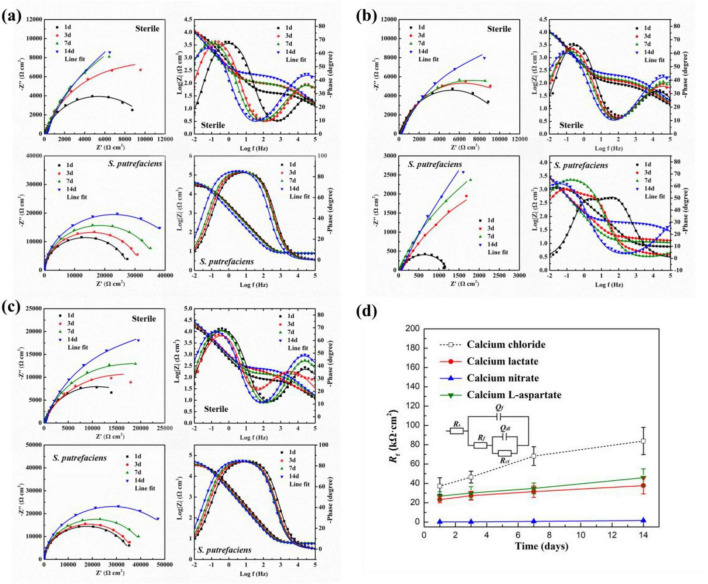
Nyquist and Bode plots of Q235 carbon steel coupons under different conditions: **(A)** calcium lactate, **(B)** calcium nitrate, and **(C)** calcium L-aspartate; **(D)** the *R*_f_ values under different conditions and the corresponding equivalent circuit.

As shown in Figure 13A, electrochemical impedance spectroscopy (EIS) results for Q235 carbon steel coupons in calcium lactate medium under both sterile and inoculated conditions at different times are presented. In the sterile medium, Nyquist plots revealed a rapid increase in the low-frequency capacitive arc radius during the first 7 days of immersion, followed by stabilization, indicating that the charge transfer process at the coupon interface was hindered. Additionally, the Bode phase angle plot showed a time constant in the high-frequency region, suggesting the formation of a product layer on the coupon surface. In inoculated media, the Nyquist plots indicated a continuous, linear increase in the low-frequency impedance modulus over time, reaching a peak at day 14. Notably, the low-frequency impedance modulus was nearly an order of magnitude higher than in the sterile group. The Bode plot in the high-frequency region displayed a broad peak in the phase angle vs. frequency curve, indicating the formation of a protective layer on the carbon steel surface. Combined with the morphology data, these results suggest that the CaCO_3_ mineralized layer formed with calcium lactate as the calcium source effectively inhibited corrosion of the carbon steel coupon.

As shown in Figure 13B, the electrochemical impedance spectroscopy (EIS) results for Q235 carbon steel coupons in calcium nitrate medium under both sterile and inoculated conditions are presented. In the sterile condition, the Nyquist plot revealed a continuous increase in the low-frequency capacitive arc radius over time. In the corresponding Bode plot, distinct peaks appeared in both the high- and low-frequency regions, indicating the formation of a protective film on the coupon surface. This film inhibited the charge transfer process at the solid-liquid interface, thereby mitigating corrosion of the Q235 carbon steel coupon. These results align with findings from studies where calcium nitrate was used as an additive to prevent concrete corrosion (Dong et al., 2018b; Justnes, 2010). Consequently, when CaCl_2_ in the 2216E medium was replaced by calcium nitrate, corrosion of Q235 carbon steel was significantly mitigated under sterile conditions. In contrast, the EIS results in the inoculated medium showed an opposite trend. The Nyquist plot indicated that the low-frequency capacitive arc radius in the inoculated medium was significantly smaller than that in the sterile medium at each time point. Although the arc radius increased slightly over time, it remained consistently smaller than in the sterile medium. In the corresponding Bode plot, the low-frequency impedance modulus reached only 3.16 × 10^3^ Ω⋅cm^2^ by day 14, indicating that the presence of *S. putrefaciens* markedly accelerates the corrosion process of the Q235 carbon steel coupon. As shown in Figure 13C, the electrochemical impedance spectroscopy (EIS) results for Q235 carbon steel coupons in calcium-L-aspartate medium under sterile and inoculated conditions are presented. In the sterile medium, the Nyquist plot showed a gradual increase in the low-frequency capacitive arc radius over time. The corresponding Bode plot revealed two distinct peaks in the phase angle curve, indicating the presence of two time constants. Notably, the low-frequency impedance modulus reached 1.51 × 10^4^ Ω⋅cm^2^ after just one day of immersion and continued to increase, reaching 2.96 × 10^4^ Ω⋅cm^2^ by day 14. In the inoculated medium, the trend in the Nyquist plot’s low-frequency capacitive arc radius was similar to that in the sterile medium, showing a steady increase over time. The Bode plot revealed a broad peak in the phase angle curve, resulting from the coupling of time constants associated with the electric double layer and the surface product film. Overall, the low-frequency impedance modulus in the inoculated medium was higher than in the sterile medium, reaching 5.02 × 10^4^ Ω⋅cm^2^ on day 14, which was 1.7 times that of the sterile medium.

As shown in Figure 13D, the changes in *R*_*f*_ values obtained from fitting the above EIS results with the equivalent circuit model. Combined with previous surface morphology data, it was evident that *S. putrefaciens* could induce the formation of carbonate deposition layers in media with various calcium sources. The *R*_*f*_ values for calcium lactate and calcium-L-aspartate showed a similar increasing trend but remained lower than those in the calcium chloride group overall. The calcium-L-aspartate group exhibited higher *R*_*f*_ values than the calcium lactate condition, potentially because aspartate ions, as anions, regulated the growth of CaCO_3_, forming a more continuous and smooth film structure (Tong et al., 2004), which aligned with previous morphological observations. In contrast, the *R*_*f*_ values for the calcium nitrate coupon’s carbonate-deposition and corrosion-product layer remained at very low levels, indicating no corrosion inhibition on the carbon steel surface.

**FIGURE 14:**
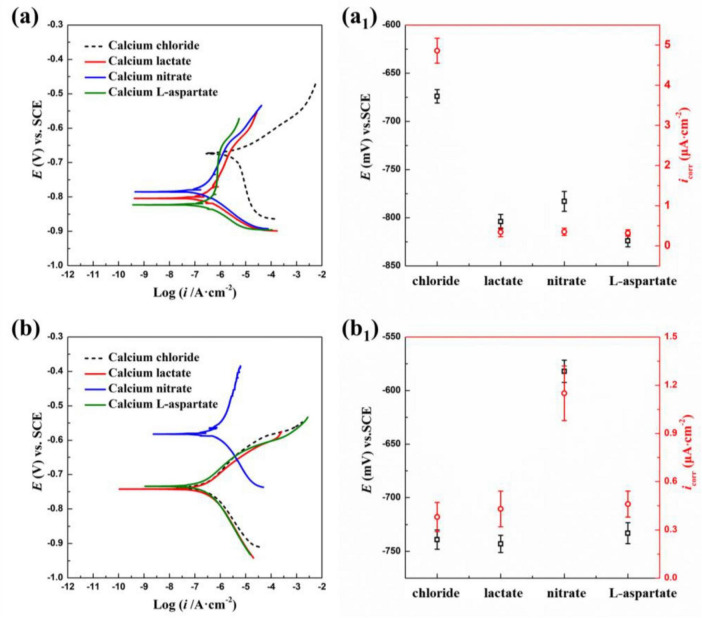
Potentiodynamic polarization curve and corresponding *E*_corr_ and *i*_corr_ analysis of Q235 carbon steel coupons soaked in sterile **(A,A_1_)** and *S. putrefaciens*-inoculated **(B,B_1_)** medium for 14 days under different calcium source conditions.

Figure 14 shows the potentiodynamic polarization curves of Q235 carbon steel coupons after 14 days of immersion in different calcium-source media under both sterile and bacterial conditions. In the sterile medium, the polarization curves shift significantly to the left relative to the standard 2216E medium, with both anodic and cathodic reactions inhibited in the calcium lactate, calcium nitrate, and calcium-L-aspartate media. As shown in Figure 14A1, the corresponding corrosion rates decrease by an order of magnitude, with similar corrosion current densities across the three calcium sources. In inoculated media (Figure 14B), the polarization curves for calcium lactate and calcium-L-aspartate resemble those of the standard 2216E medium, indicating that *S. putrefaciens* still effectively inhibits corrosion on the carbon steel surface. However, with calcium nitrate as the calcium source, the corrosion current density increases significantly, suggesting that the presence of calcium nitrate hinders the corrosion inhibition effect of *S. putrefaciens* on Q235 carbon steel.

## 4 Discussion

The above results indicate that calcium sources with different anionic compositions significantly influence the corrosion inhibition effectiveness of calcium deposition layers induced by *S. putrefaciens*. Previous studies have shown that under alkaline conditions, Ca^2 +^ ions dissociated from calcium lactate react quickly with OH^–^ to form calcium hydroxide, which mixes with early-stage corrosion products and deposits on the carbon steel surface in colloidal form (Cabrini et al., 2018). Furthermore, each calcium lactate molecule contains two lactate ions, which, due to charge delocalization, adsorb onto the carbon steel surface via their −COO^–^ groups. This adsorption competes with chloride ions, thereby slowing the corrosion process (Ormellese et al., 2009). Therefore, calcium lactate exhibits a certain degree of corrosion mitigation even under sterile conditions.

Similarly, nitrate ions also exhibit a notable corrosion inhibitory effect. Under alkaline conditions, metals typically formed stable passive films spontaneously, which protected the substrate from corrosion. Corrosive agents like Cl^–^ in the environment attacked the passive film, leading to localized corrosion. In such cases, NO_3_^–^, as a strong oxidizing agent, could rapidly re-passivate metastable corrosion sites, restoring the protective passive film on the metal surface. Additionally, NO_3_^–^ interferes with the adsorption of Cl^–^ on the surface during its reaction with the metal (Dong et al., 2018a). However, in the presence of nitrate-reducing bacteria, the effect is reversed. This phenomenon can be explained by the Biocatalytic Cathodic Nitrate Reduction (BCNR) theory proposed by Xu et al. (2013). When bacteria attach to the metal surface, their aerobic respiration nearly depletes the dissolved oxygen in the local area. Consequently, the bacteria switch to anaerobic respiration, using NO_3_^–^ as a terminal electron acceptor, which is eventually reduced to NH_4_^–^ or N_2_. The reactions involved are as follows (Dou et al., 2018):


(1) $$\text{Fe}\to {\text{Fe}}^{2+}+2{\text{e}}^{-}$$

(2) 2NO3-+10e+-12H→+N+26HO2

(3) NO3-+8e+-10H→+NH4++3HO2

As anodic dissolution reaction (1) occurs on the metal surface, the cathodic reactions (2) and (3) proceed simultaneously and combine with the anodic process, thereby accelerating the anodic reaction rate. Xu et al. (2013) calculated the Gibbs free energy changes for the coupling reactions of iron oxidation and nitrate reduction under similar conditions, yielding values of −577 and −621 kJ/mol, respectively, indicating that these processes are highly favorable from a thermodynamic perspective. Thus, in the presence of nitrate ions, while calcium deposition is observed, *S. putrefaciens* still promotes corrosion.

The corrosion inhibition mechanism of calcium-L-aspartate was similar to that of calcium lactate, as mentioned earlier. As a weak dibasic carboxylic acid, aspartic acid’s carboxyl and amino groups readily adsorbed onto the metal surface during immersion, forming an adsorption film that inhibited both anodic dissolution and Cl^–^ adsorption. In the inoculated medium, the additional aspartate ions enhanced the transamination and deamination activity of *S. putrefaciens*, producing free ammonia, which raised the medium’s pH. As shown in Figure 1B, the pH of the medium reached approximately 8.8 on day 14 under the calcium-L-aspartate condition, significantly higher than in other groups. This higher pH facilitated the formation of a stable carbonate deposition layer on the coupon surface. Notably, the calcium carbonate deposition layer formed with calcium-L-aspartate exhibited a distinct morphology compared to other conditions. Aspartate, a zwitterion with carboxyl and amino groups, selectively adsorbed onto high-energy crystal planes of calcium carbonate, inhibited their growth, and promoted low-energy planes. This selective interaction induced a gradual transition toward a more symmetrical spherical morphology (Braissant et al., 2003; Jiang et al., 2017; Takeuchi et al., 2008).

## 5 Conclusion

This study investigated the effect of *S. putrefaciens* on the corrosion inhibition behavior of Q235 carbon steel under different calcium sources. Calcium chloride in the standard 2216E marine medium was replaced by calcium lactate, calcium nitrate, and calcium-L-aspartate, while keeping the Ca^2+^ concentration constant, and immersion experiments were conducted on Q235 carbon steel. First, the growth of *S. putrefaciens* and pH values in the calcium-modified media were measured. The surface products and biofilm morphology on the steel coupons were then observed using scanning electron microscopy (SEM) and laser confocal microscopy. Electrochemical tests and surface composition analyses were also conducted to explore the microbial corrosion inhibition mechanisms of *S. putrefaciens* under different calcium conditions. The following conclusions were drawn from the experimental results:

1. *S. putrefaciens* was able to grow normally in media with calcium lactate, calcium nitrate, and calcium-L-aspartate as calcium sources, reaching the stationary phase within 3–4 days. During cultivation, the pH of all calcium-source media showed a steady increase, with the calcium-L-aspartate medium exhibiting the most pronounced pH rise, reaching over 8.5 by day 14.
2. Under sterile conditions, comparison with standard 2216E medium revealed that calcium lactate, calcium nitrate, and calcium-L-aspartate all inhibited corrosion of Q235 carbon steel, as indicated by weight loss and electrochemical tests. Mild localized corrosion was observed on coupons in calcium lactate and calcium nitrate media, while calcium-L-aspartate provided the most significant corrosion inhibition for the carbon steel coupons.
3. In the calcium lactate and calcium-L-aspartate conditions, *S. putrefaciens* formed a stable protective CaCO_3_ mineralized layer on the carbon steel surface, significantly inhibiting corrosion. The aspartate ion’s regulation of CaCO_3_ crystal growth resulted in a more continuous and smoother mineralized layer.
4. With calcium nitrate as the calcium source, *S. putrefaciens* induced carbonate deposition but failed to form a continuous protective mineralized layer on the carbon steel surface. Furthermore, the biocatalytic nitrate reduction process accelerated the corrosion rate of Q235 carbon steel. Cross-sectional morphology and compositional analysis indicated that uneven mineralized particles and corrosion product accumulation led to localized corrosion.

## Data Availability

The raw data supporting the conclusions of this article will be made available by the authors, without undue reservation.
